# The clinical importance of metagenomic next-generation sequencing in detecting disease-causing microorganisms in cases of sepsis acquired in the community or hospital setting

**DOI:** 10.3389/fmicb.2024.1384166

**Published:** 2024-04-15

**Authors:** Dan Zhang, Xingxing Li, Yu Wang, Yong Zhao, Hong Zhang

**Affiliations:** Department of Emergency Medicine, The First Affiliated Hospital of Anhui Medical University, Anhui, Hefei, China

**Keywords:** community-acquired sepsis, hospital-acquired sepsis, mNGS, microorganisms, optimizing antimicrobial therapy, medical expenses

## Abstract

**Objectives:**

Although metagenomic next-generation sequencing (mNGS) is commonly used for diagnosing infectious diseases, clinicians face limited options due to the high costs that are not covered by basic medical insurance. The goal of this research is to challenge this bias through a thorough examination and evaluation of the clinical importance of mNGS in precisely identifying pathogenic microorganisms in cases of sepsis acquired in the community or in hospitals.

**Methods:**

A retrospective observational study took place at a tertiary teaching hospital in China from January to December 2021. Data on 308 sepsis patients were collected, and the performance of etiological examination was compared between mNGS and traditional culture method.

**Results:**

Two hundred twenty-nine cases were observed in the community-acquired sepsis (CAS) group and 79 cases in the hospital-acquired sepsis (HAS) group. In comparison with conventional culture, mNGS showed a significantly higher rate of positivity in both the CAS group (88.21% vs. 25.76%, adj.*P* < 0.001) and the HAS group (87.34% vs. 44.30%, adj.*P* < 0.001), particularly across various infection sites and specimens, which were not influenced by factors like antibiotic exposure or the timing and frequency of mNGS technology. Sepsis pathogens detected by mNGS were broad, especially viruses, *Mycobacterium tuberculosis*, and atypical pathogens, with mixed pathogens being common, particularly bacterial-viral co-detection. Based on the optimization of antimicrobial therapy using mNGS, 58 patients underwent antibiotic de-escalation, two patients were switched to antiviral therapy, and 14 patients initiated treatment for tuberculosis, resulting in a reduction in antibiotic overuse but without significant impact on sepsis prognosis. The HAS group exhibited a critical condition, poor prognosis, high medical expenses, and variations in etiology, yet the mNGS results did not result in increased medical costs for either group.

**Conclusions:**

mNGS demonstrates efficacy in identifying multiple pathogens responsible for sepsis, with mixed pathogens of bacteria and viruses being prevalent. Variability in microbiological profiles among different infection setting underscores the importance of clinical vigilance. Therefore, the adoption of mNGS for microbiological diagnosis of sepsis warrants acknowledgment and promotion.

## Introduction

Sepsis, a prevalent and severe clinical condition characterized by organ dysfunction resulting from immune response dysfunction caused by infection, has garnered significant attention worldwide due to its substantial health risks and financial burden (Singer et al., 2016; Rhodes et al., 2017; Buchman et al., 2020). In 2017, the World Health Organization (WHO) introduced a resolution stressing the importance of improving the recognition, diagnosis, treatment, and prevention of sepsis due to the pressing need for emergency care (WHO, 2017). The management of sepsis treatment is complicated due to the wide range of pathogenic infections, the challenge of promptly identifying pathogens, and the need for precise treatment in the early stages. While culture has long been viewed as the benchmark for diagnosing sepsis, it does have some drawbacks. These include the time-consuming nature of the process, which can take 2–7 days, a low rate of positive results, susceptibility to contamination, a limited spectrum of pathogen detection, and vulnerability to the influence of antibiotics. These constraints may lead to delayed treatment with antibacterial drugs, excessive use of broad-spectrum antibiotics, a rise in resistance to microbial drugs, and increased medical costs (Miao et al., 2018). Hence, the identification and prompt detection of pathogens without the aforementioned issues necessitate the exploration of a technology. Researchers have shown growing interest in metagenomic next-generation sequencing (mNGS) technology in this particular situation.

mNGS employs high-throughput gene sequencing technology to simultaneously detect the deoxyribonucleic acid (DNA) or ribonucleic acid (RNA) of all microorganisms present in clinical samples, enabling the determination of potential pathogenic microorganism types through database comparison and bioinformatics analysis (Sharon and Banfield, 2013; Chiu and Miller, 2019). It has the characteristics of no culture, no dependence on specific sequence amplification, no bias, less time consumption, high sensitivity, not affected by a variety of bacterial species and antibiotic treatment, which is widely used in infectious diseases (Ishihara et al., 2020; Gu et al., 2021; Miller and Chiu, 2022; Yang et al., 2022). Prior research have shown that mNGS results obtain higher positive rate and clinical coincidence rate in sepsis pathogen detection, which is helpful for medical decision-making, optimizing antibiotic management and improving prognosis (Wang et al., 2023; Zuo et al., 2023). Clinicians frequently do not prioritize utilizing mNGS due to its expensive nature and limited coverage by health insurance.

Research found that community-acquired sepsis (CAS) makes up around 70% of sepsis cases (Reinhart et al., 2017), with distinct pathogen characteristics, treatment strategies, and survival rates among CAS and hospital-acquired sepsis (HAS) (Tonai et al., 2022; Kim et al., 2023). While timely administration of effective antibiotics is crucial in reducing sepsis mortality, the significance of pathogen culture should not be overlooked (Niederman et al., 2021). Given the limited exploration of the disparities between CAS and HAS in existing literature, further investigation into the utility of mNGS in these contexts is warranted. Therefore, the aim of this research was to evaluate the medical importance of mNGS in detecting pathogens and offering treatment recommendations for sepsis acquired in the community or in a hospital setting.

## Materials and methods

### Study participants and groups

A retrospective observational study on sepsis was conducted at a single center, using analytical cross-sectional cohort methods. Between January 2021 and December 2021, the First Affiliated Hospital of Anhui Medical University enrolled 308 participants. Patients were selected if they met specific criteria, including the third international consensus diagnostic criteria for sepsis and septic shock (Sepsis-3) from 2016, with a Sequential Organ Failure Assessment (SOFA) score of ≥2, being over 18 years old, providing consent for mNGS examination, having complete clinical and laboratory data available, and undergoing both mNGS and conventional culture methods for examination. Participants who did not meet the study criteria were excluded. This included individuals under 18 years old, those who refused mNGS examination, pregnant or lactating individuals, and those with incomplete or insufficient clinical data.

Patients were categorized into two groups, namely, the CAS group and the HAS group, based on the location of onset as documented in the clinical electronic medical record system. The CAS group consisted of patients who developed sepsis due to infections acquired prior to hospitalization, as well as those who exhibited symptoms within 48 h of hospitalization during the incubation period. In contrast, sepsis caused by the aforementioned infections was classified as HAS if it met the following criteria: (1) Infections without an indeterminate incubation period beyond 48 h of admission; (2) Infections with a clearly defined incubation period that exceeded the average incubation period upon admission; (3) Infections directly associated with the most recent hospitalization; (4) Occurrence of new infections in other anatomical sites based on the original infection; (5) Identification of new pathogens in addition to known pathogens from the original infection. Based on the findings from mNGS, sepsis was divided into two categories: those with positive mNGS results and those with negative mNGS results. Utilize a comprehensive approach that incorporates mNGS and traditional culture findings, clinical presentation, inflammatory markers, and imaging studies to tailor and refine therapeutic interventions.

### Clinical information collection

Data collected from the electronic medical record system includes a range of clinical details. These details encompass demographic information, medical history, infection location, SOFA score, acute physiology and chronic health evaluation II (APACHE II) score, length of hospital stay, intensive care unit (ICU) admission, ICU length of stay, treatments (like vasoactive drugs, mechanical ventilation, and renal replacement therapy), and mortality rate. Furthermore, the laboratory results included various blood parameters such as white blood cell (WBC) count, neutrophils, lymphocytes, neutrophil to lymphocyte ratio (NLR), red blood cell (RBC) count, hemoglobin, and platelets. Additionally, levels of total bilirubin (TBIL), alanine aminotransferase (ALT), aspartate aminotransferase (AST), prothrombin time (PT), activated partial thrombin time (APTT), prothrombin activity (PTA), fibrinogen, d-dimer (D-D), albumin, blood urea nitrogen (BUN), serum creatinine (Scr), lactic acid (Lac), and inflammatory markers like C-reactive protein (CRP), and procalcitonin (PCT) were recorded. The factors to be considered in this study also include the administration of antibiotics, the detecting timing and frequency of the mNGS technique, adjustment of antimicrobial drugs, and various medical costs. These costs consist of total hospitalization fees, average daily hospitalization fees, diagnosis costs (including laboratory diagnosis costs and clinical diagnosis project fees), integrated medical service costs (comprising medical service fees, treatment operation fees, nursing fees, and operation fees), as well as treatment costs (including western medicine fees, antibacterial drug fees, Chinese patent medicine fees, and blood fees), and consumables expenses.

### Microbiological analyses

Samples from various infection sites of sepsis, including blood, sputum, bronchoalveolar lavage fluid (BALF), urine, cerebrospinal fluid (CSF), pleural effusion, ascites, pus, tissue, hydropericardium, and bone marrow, were collected in accordance with National Clinical Laboratory Procedures (Shang, 2015). These samples were expeditiously transferred to the microbiology laboratory and mNGS Laboratory at the First Affiliated Hospital of Anhui Medical University for Standard Microbial Culture (Shang, 2015) and mNGS detection procedures (Lu and Wang, 2020). Various pathogens were identified through microbial cultivation and automatic analysis in the microbiology laboratory. Blood culture was emphasized, with each group necessitating two culture bottles, one aerobic and one anaerobic. In cases where the infection site was ambiguous or the specimen was unattainable, a blood specimen was chosen, and the culture protocol was adjusted according to the suspected infection site. Performing all culture types for each patient was deemed unnecessary.

### Metagenomic next-generation sequencing experiments and data analysis

In accordance with the manufacturer's guidelines, samples were processed to extract and purify DNA utilizing the QIAamp DNA Micro Kit (QIAGEN, Hilden, Germany). Subsequent DNA library construction was completed using the Qiagen library construction kit (QIAseq Ultralow Input library kit). Quality assessment of the library was performed using the Qubit 3.0 Fluorometer (Invitrogen, Q33216) and Agilent 2100 Bioanalyzer (Agilent Technologies, Palo Alto, USA). Following this, sequencing was conducted on the Illumina Nextseq 550 sequencing platform (Illumina, San Diego, USA) with SE75bp sequencing strategy. The data underwent quality filtering to remove adapters, low-quality, low-complexity, and short sequences, followed by the utilization of Scalable Nucleotide Alignment Program (SNAP; v2.0.1) software to eliminate human-derived sequences aligned with the human reference database (hg38). Subsequently, the non-human data were classified by simultaneous alignment to the reference microbial sequences from bacteria, viruses, fungi, which were obtained from the NCBI Nucleotide database (https://ftp.ncbi.nlm.nih.gov/genomes/) (Chiu and Miller, 2019). Sequence alignment was performed by BLASTN (v2.11.0+) with “megablast” option, and only reads uniquely aligning to microbial taxa were tallied. The final microbial identification results for the samples were then determined. Using peripheral blood samples from healthy donors as negative controls and sterile deionized water as non-template controls. Reads per million mapped reads (RPM) was defined as the number of reads of target pathogen per million of total filtered reads. The identification of positive criteria is not reliant on any singular indicator, including but not limited to the number of identified sequences for particular microorganisms, the ratio of normalized RPM, or the genome coverage of detected species. The formula used to determine the normalized RPM of the pathogen is expressed as follows: RPM of the pathogen = (number of reads mapped to the pathogen × 10^6^)/(total number of mapped reads from the given library) (Li et al., 2023b). For bacteria other than *Mycobacterium tuberculosis*, fungi other than *Cryptococcus*, and parasites, identification was based on sequencing coverage ranking within the top 10 of all detected pathogens and the absence in the negative control (NTC), or a sample/NTC RPM ratio exceeding 10. Conversely, for viruses, *M. tuberculosis*, and Cryptococci, identification relied on the presence of at least one specific sequence not found in the NTC, or a sample/NTC RPM ratio >5 (Zhang et al., 2023).

### Statistical analysis

The data were analyzed and graphed using GraphPad-Prism 9. Continuous variables were depicted as mean ± standard deviation (SD) for normally distributed data and as median (25th percentile, 75th percentile) for non-normally distributed data. Inter-group comparisons for continuous variables were conducted using independent sample *t*-tests or non-parametric tests. Categorical variables were expressed as numerical values and percentages, and evaluated through chi-square or Fisher's exact test. *P*-values were adjusted using the Holm-Sidak method. G^*^Power software was used to calculate sample size and statistical testing power. A significance level of adjusted *P* (adj.*P*) value < 0.05 was utilized to determine statistical significance.

## Results

### Baseline characteristics of study participants

Table 1 shows the distribution of baseline characteristics of 308 participants between CAS and HAS, including clinical features, past history, treatments, and mortality rate. Of the 308 participants, 74.35% (229/308) belonged to CAS, while 25.65% (79/308) belonged to HAS. The median days of admission were 22 for CAS and 32 for HAS, with a significant difference (adj.*P* = 0.0003). The median age for both groups was similar, with 58 years for CAS and 57 years for HAS (adj.*P* = 0.7876). The medical histories of the two cohorts of patients encompass a range of conditions such as hypertension, diabetes, heart disease, neurogenic disease, chronic renal insufficiency, chronic obstructive pulmonary disease, and immune-related diseases, etc. Analysis of Table 1 reveals no statistically significant difference in medical history between the two groups (adj.*P* > 0.05). Additionally, the severity of the patient's condition was assessed using SOFA scores and APACHE II scores, which indicated that HAS had a higher severity. Furthermore, HAS had a higher ICU admission rate, longer hospitalization time, received more vasoactive drugs and mechanical ventilation, and required more glucocorticoid therapy, and blood products therapy. HAS had a higher likelihood of developing multiple organ failure and a slightly increased mortality rate. However, there was no statistical difference in mortality rate compared to CAS.

**Table 1:** The baseline characteristics of participants.

**Clinical feature**	**CAS group**	**HAS group**	***P*-value**	**adj. *P***
Male *N* (%)	62.01% (142/229)	67.09% (53/79)	0.4192	0.6627
Age (years)	58 (48–69.50)	57 (45–68)	0.7876	0.7876
SOFA score	5 (3–9)	8 (5–12)	**< 0.0001**	**0.0004**
APACHE II score	14 (9–21)	18 (12–23)	**0.0065**	**0.0321**
Length of admission (day)	22 (13–35.50)	32 (25–42)	**< 0.0001**	**0.0003**
ICU admission (%)	43.23% (99/229)	67.09% (53/79)	**0.0003**	**0.0018**
ICU length of stay (day)	16 (9–24)	19 (13–31)	**0.0413**	0.1552
**Past history**				
Hypertension (%)	37.99% (87/229)	39.24% (31/79)	0.8439	0.9672
Diabetes (%)	16.16% (37/229)	20.25% (16/79)	0.4056	0.9390
Cardiopathy (%)	9.61% (22/229)	21.52% (17/79)	**0.006**	0.0584
Neurogenic disease	10.48% (24/229)	20.25% (16/79)	**0.0259**	0.2104
Chronic kidney dysfunction (%)	10.04% (23/229)	6.33% (5/79)	0.3735	0.9390
Chronic obstructive pulmonary disease (%)	9.17% (21/229)	7.59% (6/79)	0.819	0.9672
Immune-related diseases (%)	28.82% (66/229)	32.91% (26/79)	0.5687	0.9390
Smoking	14.41% (33/229)	8.86% (7/79)	0.2472	0.8630
Drinking	10.48% (24/229)	6.33% (5/79)	0.3726	0.9390
No underlying diseases	26.20% (60/229)	16.46% (13/79)	0.0919	0.5375
**Treatments**				
Vasoactive drug therapy (%)	39.30% (90/229)	68.35% (54/79)	**< 0.0001**	**0.0004**
Mechanical ventilation (%)	37.12% (85/229)	62.03% (49/79)	**0.0001**	**0.0005**
Duration of mechanical ventilation (day)	11 (7–20)	15 (10.50–22.50)	0.0729	0.2031
Renal replacement therapy (%)	15.72% (36/229)	24.05% (19/79)	0.1242	0.2330
Duration of renal replacement therapy (hour)	87 (40.63–205.2)	64 (40–128.5)	0.363	0.3630
Glucocorticoid therapy (%)	63.32% (145/229)	81.01% (64/79)	**0.0034**	**0.0135**
Blood products therapy (%)	68.56% (157/229)	97.47% (77/79)	**< 0.0001**	**0.0001**
Case fatality rates	26.64% (61/229)	36.71% (29/79)	0.114	0.3045

CAS, community-acquired sepsis; HAS, hospital-acquired sepsis; SOFA, sequential organ failure assessment; APACHE II, acute physiology and chronic health evaluation II; ICU, intensive care unit; adj.*P*, Adjusted *P*-value. The bold values indicate statistical significance.

### Analysis of laboratory data among the CAS and HAS groups

When examining the correlation between laboratory results in CAS and HAS groups during sepsis diagnosis, it was discovered that the HAS group had lower levels of RBC, hemoglobin, and PTA compared to the CAS group (all adj.*P* < 0.05). Furthermore, the levels of BUN and PT were elevated in the HAS group compared to the CAS group, with statistically significant differences observed (all adj.*P* < 0.05). The results suggest that people in the HAS category had a higher likelihood of experiencing anemia, impaired blood clotting, and damage to kidney function (see Supplementary Table 1).

### Comparison clinical diagnostic outcome of mNGS and traditional culture

**Figure 1:**
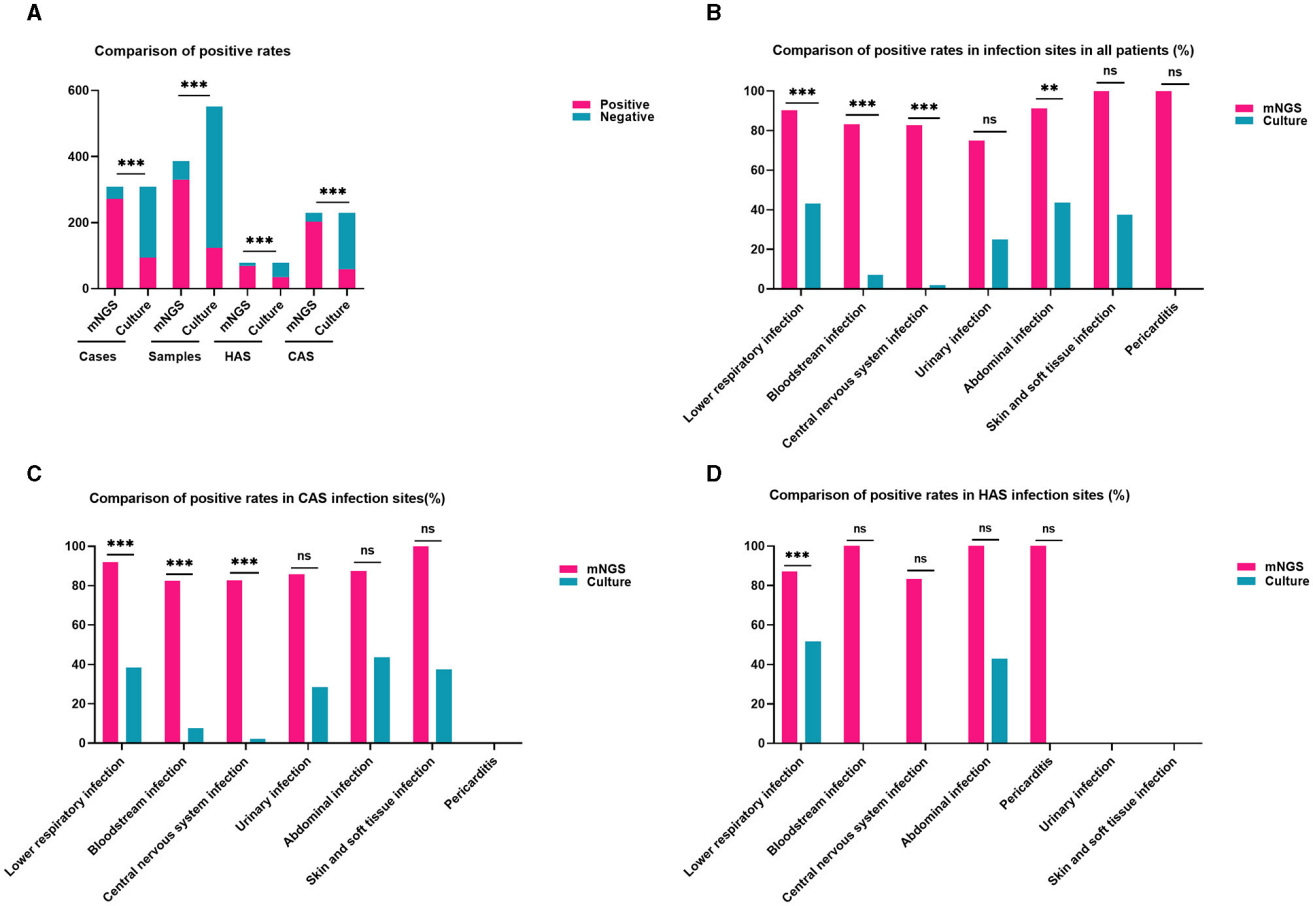
Comparison of positivity rates between metagenomic next-generation sequencing (mNGS) and traditional culture methods. **(A)** The positive rates of mNGS outweigh the culture method in all cases, samples, HAS, and CAS (all adj.*P* < 0.001). **(B)** The detection rate of mNGS in different infection locations was higher compared to culture in all patients, particularly in cases of lower respiratory infections, bloodstream infections, central nervous system infections, and abdominal infections (all adj.*P* < 0.01). **(C)** In the CAS group, mNGS showed higher positivity rates than culture in lower respiratory infections, bloodstream infections, and central nervous system infection sites were higher than in culture (all adj.*P* < 0.001). **(D)** The detection rate of mNGS in the lower respiratory tract was higher than that of culture in the HAS group (adj.*P* < 0.001). Significance levels: ^**^P < 0.01; ^***^P < 0.001; ns, no statistically significant variation.

The study found that the detection rates of mNGS and traditional culture were 87.99% (271/308) and 30.52% (94/308) in every instance. There were 387 mNGS specimens and 552 culture specimens collected for mNGS and culture detection, with positive rates of 85.27% (330/387) and 22.28% (123/552), respectively. Certainly, the mNGS positive rate was increased by almost 60% in both cases and samples compared to traditional culture, a statistically significant difference shown in the Chi-squared test of positive rate (adj.*P* < 0.001). Moreover, within the CAS cohort, the detection rate of mNGS (202/229, 88.21%) was approximately 63% greater than that of conventional culture (59/229, 25.76%), showing a statistically significant disparity (adj.*P* < 0.001). Similarly, the HAS group also showed comparable results [(69/79, 87.34%) vs. (35/79, 44.30%), adj.*P* < 0.001; Figure 1A]. These findings suggest that the detection rates of mNGS were similar in the CAS and HAS groups, and both groups had significantly higher detection rates compared to traditional culture. Further study showed that 90 patients were positive for mNGS and traditional culture, and the coincidence rates of mNGS and traditional culture in detecting the same pathogen were 81.03% (47/58) for CAS and 84.35% (27/32) for HAS, respectively.

**Figure 2:**
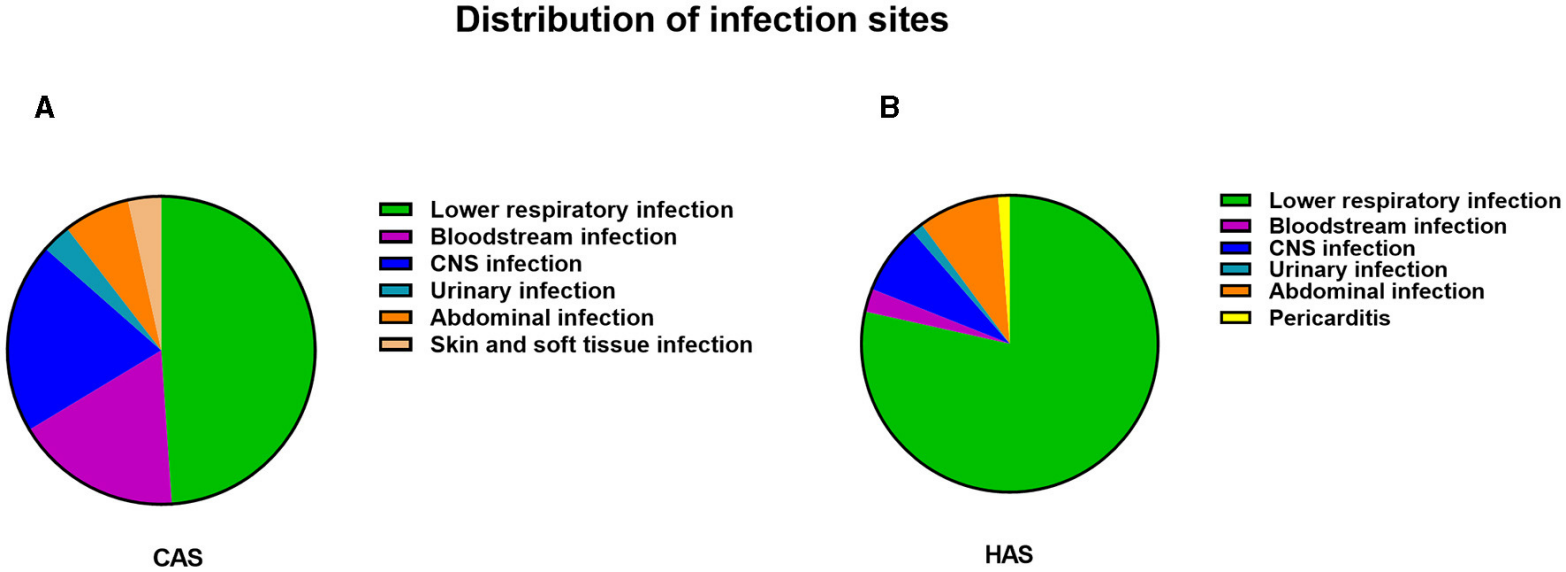
The distribution of infection in CAS group and HAS group. **(A)** Distribution of infection sites in CAS group. **(B)** Distribution of infection sites in HAS group. Lower respiratory tract infections were found to be the predominant site of infection, regardless of whether the patients were in the CAS or HAS group.

The distribution of infection sites in this study is shown in Figure 2. Obviously, lower respiratory infection was the most common in both the CAS group and HAS group. In each infection site, the positive rate of mNGS was higher than that of culture, as shown in Figure 1B, particularly in lower respiratory infection (adj.*P* < 0.001), bloodstream infection (adj.*P* < 0.001), central nervous system (CNS) infection (adj.*P* < 0.001), and abdominal infection (adj.*P* < 0.01). However, the disparity in cases of urinary infection, skin and soft tissue infection, and pericarditis did not reach statistical significance due to the limited sample size. Similar results were also seen in the CAS group (Figure 1C). Nevertheless, within the HAS cohort, notable variances in detection rates were solely noted in lower respiratory (Figure 1D).

**Figure 3:**
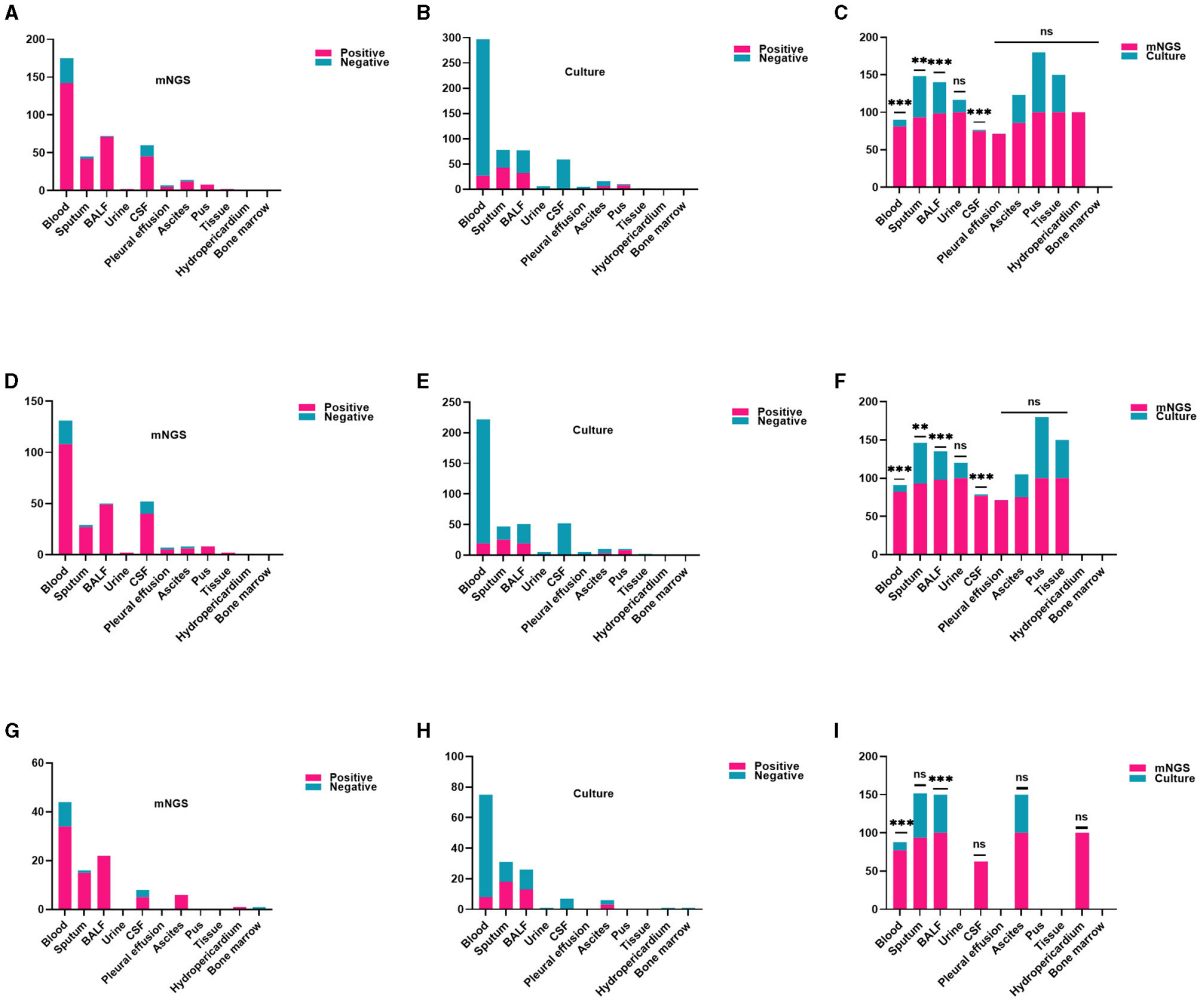
Comparison of the positive rate of mNGS and traditional culture in different samples. **(A–C)** In every patient, the detection rate of mNGS in each specimen was notably higher compared to culture, particularly in blood, sputum, BALF, CSF (all adj.*P* < 0.01). **(D–F)** Within the CAS cohort, the detection rate of mNGS was significantly higher than that of traditional culture for various sample types including blood, sputum, BALF, and CSF (all adj.*P* < 0.01). **(G–I)** In the HAS group, mNGS results showed a higher positive rate in blood and BALF (all adj.*P* < 0.01). CSF, cerebrospinal fluid; BALF, bronchoalveolar lavage fluid. Significance levels: ^**^P < 0.01; ^***^P < 0.001; ns, no statistically significant variation.

Figure 3 shows the distribution of assorted samples. The most common mNGS specimen was blood (175/387, 45.22%), followed by BALF (72/387, 18.60%), CSF (60/387, 15.50%), sputum (45/387, 11.63%), ascites (14/387, 3.62%), and pus (8/387, 2.07%), pleural effusion (7/387, 1.81%), urine (2/387, 0.52%), skin and soft tissue (2/387, 0.52%), pericardial effusion (1/387, 0.26%), and bone marrow (1/387, 0.26%) in all cases. These results demonstrate a notably higher positive rate of mNGS compared to culture, particularly in blood (adj.*P* < 0.001), sputum (adj.*P* < 0.01), BALF (adj.*P* < 0.001), CSF (adj.*P* < 0.001; Figures 3A–C). The CAS group found similar results in subtypes of blood (adj.*P* < 0.001), sputum (adj.*P* < 0.01), BALF (adj.*P* < 0.001), and CSF (adj.*P* < 0.001; Figures 3D–F). Likewise, similar findings were observed in blood, and BALF in the HAS group, showing statistically significant variances (Figures 3G–I).

### Comparison of pathogenic characteristics by mNGS and traditional culture

**Figure 4:**
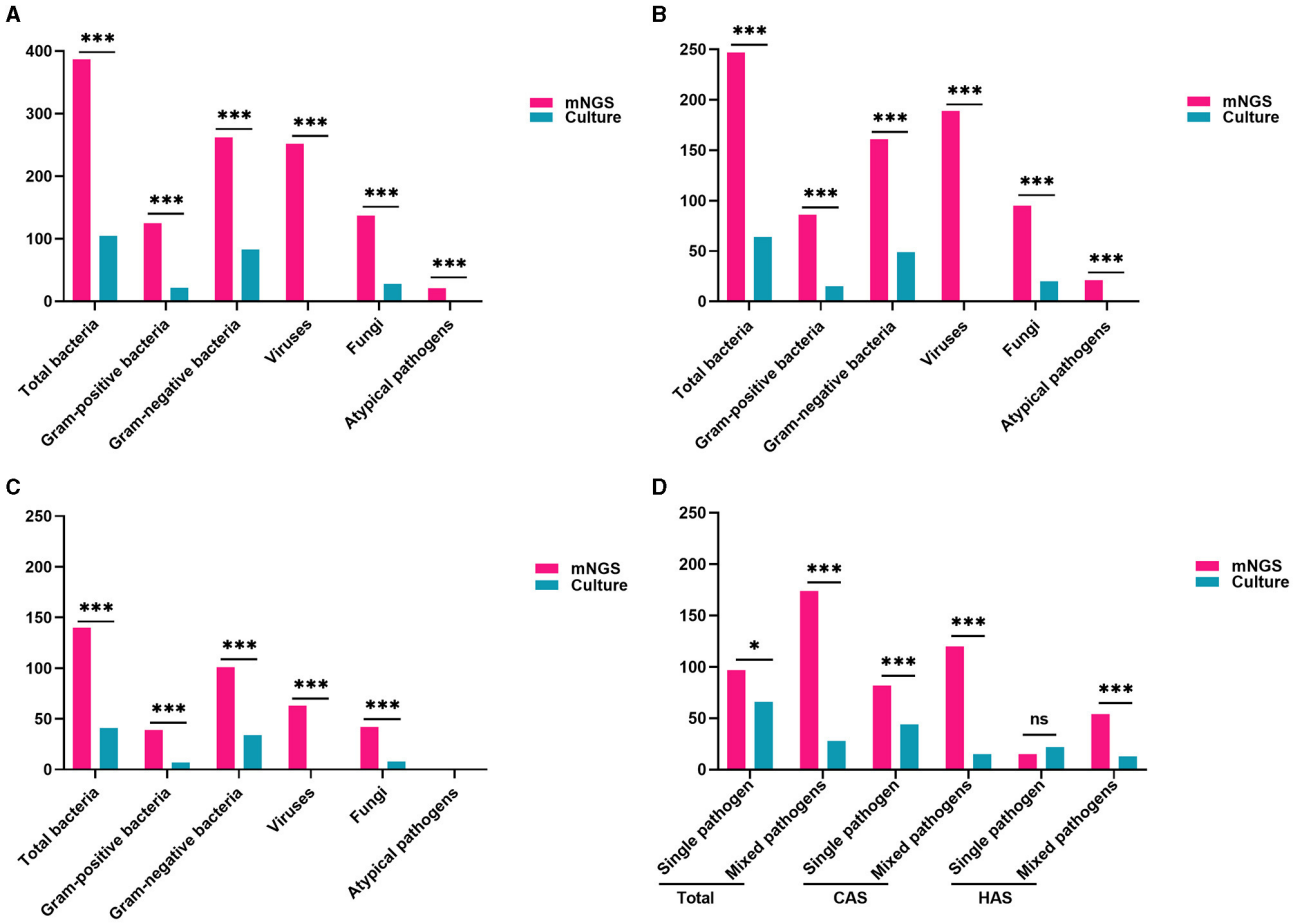
Comparison of pathogen traits identified by mNGS and conventional culture methods. **(A)** Comparison of pathogen types and positive rate in all patients (all adj.*P* < 0.001). **(B)** Comparison of pathogen types and positive rate in CAS group (all adj.*P* < 0.001). **(C)** Comparison of pathogen types and positive rates in the HAS group (all adj.*P* < 0.001, except for atypical pathogens). **(D)** mNGS was more common for mixed pathogen infections, while traditional culture was more frequent for single pathogen detection. With the exception of identifying a single pathogen in the HAS group, the positive rate of mNGS significantly surpassed that of traditional culture (all adj.*P* < 0.05). Significance levels: ^*^P < 0.05; ^***^P < 0.001; ns, no statistically significant variation.

In this study, mNGS detected 797 pathogens, including 387 bacteria, 252 viruses, 137 fungi, and 21 atypical pathogens. The detection rates of bacteria and fungi using mNGS were markedly superior to those achieved through traditional culture methods (all adj.*P* < 0.001). Regarding the examination of bacteria, the detection rates of mNGS and conventional culture for gram-negative bacteria were notably greater than those for gram-positive bacteria (all adj.*P* < 0.001). Moreover, mNGS identified viruses and unusual pathogens that are undetectable through conventional methods, demonstrating its distinct advantages (Figure 4A, all adj.*P* < 0.001). Similar findings were observed in the CAS and HAS groups, as shown in Figures 4B, C. Positive results from mNGS for different pathogens were significantly higher in both groups than traditional culture methods (all adj.*P* < 0.001). However, it is important to note that atypical pathogens were exclusively identified through mNGS only in the CAS group.

The research also found that mNGS detection of mixed pathogens named after two or more pathogens was more common (174/271, 64.21%), while traditional culture was more frequently used for single pathogen (65/94, 69.15%). In both the total cases and CAS, the positive rates of mNGS significantly exceeded that of traditional culture, whether it was for single pathogen or mixed pathogens (all adj.*P* < 0.05). Similarly, mNGS was superior to traditional culture in detecting mixed pathogens in the HAS group (adj.*P* < 0.001). Nevertheless, when identifying a single pathogen, there was no statistically significant variance found between the two techniques (Figure 4D, adj.*P* = 0.2595). The findings clearly show that the detection rate of mixed pathogens using mNGS was significantly higher than that of single pathogens across different subgroups (adj.*P* < 0.001 for overall cases, adj.*P* < 0.001 for CAS, adj.*P* < 0.001 for HAS).

### Analysis of pathogens detected by mNGS and traditional culture

**Figure 5:**
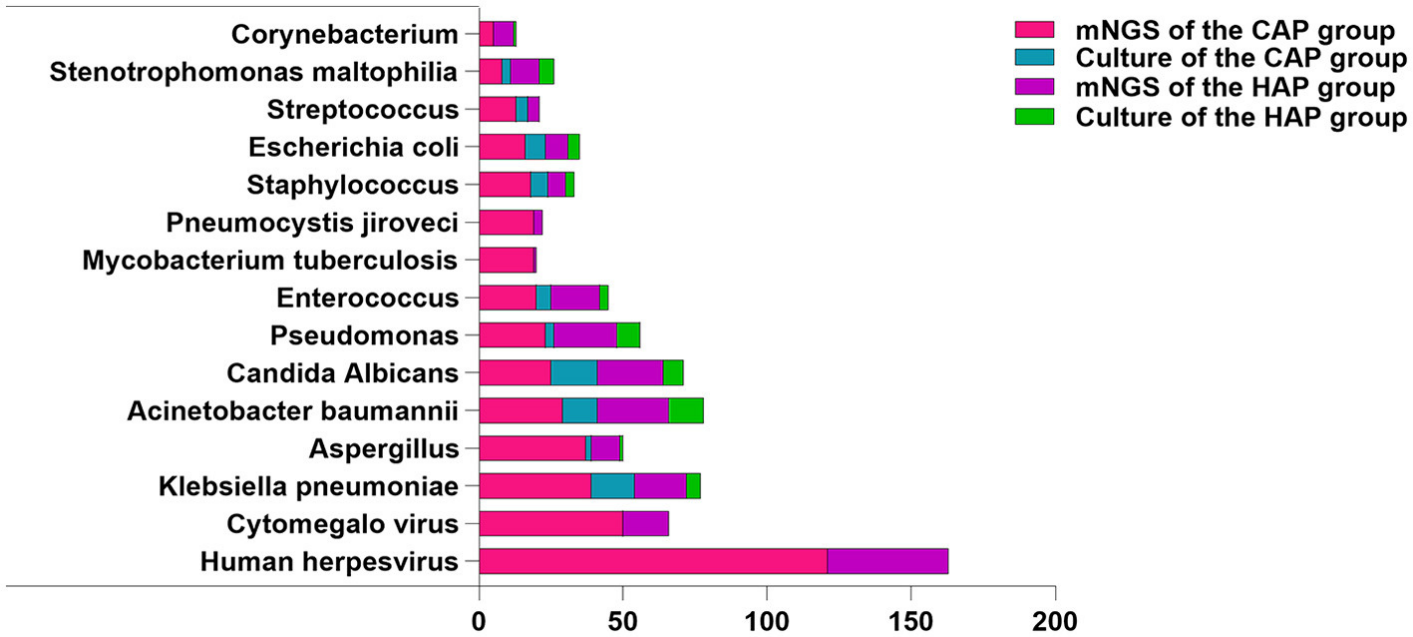
Distribution of pathogen species detected by mNGS and culture. Tested pathogens are represented on the *X*-axis by their counts.

Subsequently, we further studied the common pathogen types in the CAS and HAS groups. In the CAS group, Human herpesvirus (*n* = 121) was found to be the most hackneyed among the top 10 pathogens tested by mNGS, while *Klebsiella pneumoniae* (*n* = 39), and *Aspergillus* (*n* = 37) were the most frequent bacteria and fungi. However, only bacteria and fungi were detected in traditional cultures, with *K. pneumoniae* (*n* = 15) and *Candida albicans* (*n* = 16) being the commonest. Additionally, mNGS detected 19 cases of *M. tuberculosis*, demonstrating advantages beyond traditional culture. In the HAS group, Human herpesvirus (*n* = 42) remained the most frequently identified pathogen using mNGS, although the bacteria and fungi detected differed compared to those found in the CAS group. The most frequently bacteria were *Acinetobacter baumannii* (*n* = 25), and the fungi detected were *C. albicans* (*n* = 23). Similarly, the most frequently found bacteria and fungi identified through conventional culture methods were comparable to those identified through mNGS. Nevertheless, whether in the CAS group or the HAS group, the rates of bacterial and fungal detection by traditional culture were significantly lower compared to mNGS. The top 10 specific pathogen is illustrated in Figure 5. Following that, we conducted a detailed analysis of prevalent Gram-positive bacteria (top 5), Gram-negative bacteria (top 5), fungi (top 5), various viruses, and atypical pathogens. For further information, please consult Supplementary Figure 1A.

### Comparison of pathogen types between single pathogen and mixed pathogens

Next, we conducted an etiological analysis at the level of single pathogen and mixed pathogens. Regarding the single pathogen identified through the aforementioned methodologies, mNGS revealed that bacteria were the predominant pathogen in the CAS group, followed by viruses, fungi, and atypical pathogens. However, traditional culture methods solely detected bacteria and fungi. In the HAS group, mNGS predominantly identified bacteria, followed by fungi and viruses, with no atypical pathogens detected. Conversely, traditional culture methods exhibited limited detection of pathogenic types, with bacteria being the most frequently identified.

This study demonstrates a higher prevalence of mixed pathogens in the etiology of sepsis. The utilization of mNGS revealed that bacterial-viral co-detection were more frequently observed in both the CAS and HAS groups, whereas traditional culture methods indicated a higher incidence of bacterial-fungal co-infections specifically in the CAS group. Conversely, multiple bacterial infections were more commonly observed in the HAS group. These findings highlight that the single pathogen commonly found in mNGS-based pathogen detection in sepsis is bacteria, and bacterial-viral co-detection is the most prevalent form of mixed pathogens (Table 2). For specific common bacterial strains, please refer to Supplementary Figures 1B–D.

**Table 2:** Comparison of types between single pathogen and mixed pathogens.

**Group/type**	**CAS group**		**HAS group**	
	**mNGS**	**Culture**	**mNGS**	**Culture**
Single pathogen	82	44	15	22
Bacteria	40	35	9	20
Fungi	11	9	4	2
Viruses	27		2	
Atypical pathogens	4			
Mixed pathogens	120	15	54	13
Multiple bacteria	10	6	11	9
Multiple viruses	5		3	
Multiple fungi	1	1		1
Bacteria combined with fungi	13	8	9	3
Bacteria combined with viruses	41		15	
Bacteria combined with atypical pathogens	2			
Viruses combined with atypical pathogens	7			
Viruses combined with fungi	10		4	
Bacteria, viruses, and fungi	26		12	
Bacteria, viruses, and atypical pathogens	3			
Viruses, fungi, and atypical pathogens	1			
Bacteria, viruses, fungi, and atypical pathogens	1			

### Analyze the impact of antibiotic exposure, timing and frequency of **mNGS** testing on mNGS results

The purpose of this research was to investigate how antibiotics exposure, timing, and frequency of mNGS testing could affect the results of mNGS. The results showed that conducting multiple mNGS tests in individuals with CAS and HAS led to a higher rate of positive results compared to a single test, although this discrepancy was not statistically significant (all adj.*P* > 0.05). Furthermore, the positive rate of mNGS was not affected by antibiotics exposure or the timing of mNGS detection, regardless of patients' group (CAS or HAS). Additionally, it was noted that the mortality rate was elevated in the mNGS positive group when compared to the mNGS negative group within both the CAS and HAS groups. It is important to mention that there was no statistically significant distinction between the two groups, as shown in Supplementary Table 2.

### Optimizing antimicrobial therapy based on mNGS and culture results

This study examined the optimization of antimicrobial therapy for sepsis by utilizing both mNGS and culture results. In contrast to the clinical manifestations, inflammatory markers, routine culture findings, and therapeutic outcomes of sepsis, the positive mNGS results in 235 cases demonstrated a favorable influence on clinical management. These findings contribute to the elucidation of pathogenic diagnosis and can be regarded as a well-matched clinical diagnostic group. Conversely, the mNGS results of 73 cases did not exert any discernible impact on clinical treatment. Out of these, 36 cases were positive for mNGS but deemed to be mismatched with the clinical diagnosis group, while 37 cases were negative for mNGS. In the CAS group, there were 126 cases where antimicrobial therapy was optimized based on mNGS and traditional culture results. Among these cases, 41 cases had down-regulated antibiotics, two cases switched to antiviral treatment, and 14 cases switched to anti-tuberculosis therapy. Additionally, within the cohort of patients in the HAS group, a total of 48 mNGS positive patients underwent optimization of their anti-infection regimens, with 17 cases having antibiotic treatment down-regulated. Nevertheless, in the mismatch clinical diagnosis group, there were six cases where the antibiotic regimen was optimized using traditional culture results, of which one case was down-regulated in the CAS and HAS groups, respectively (Table 3).

**Table 3:** Optimizing antimicrobial therapy and mortality rate analysis based on mNGS and culture results.

**Project**	**CAS group**		**HAS group**		***P*-value**	**adj.*P***
	**Number**	**Mortality rate**	**Number**	**Mortality rate**		
**mNGS positive**						
Match clinical diagnosis	169	27.81% (47/169)	66	40.91% (27/66)	0.0612	0.5313
Adjusting treatment	126	30.95% (39/126)	48	45.83% (22/48)	0.0767	0.5843
Adjustment by mNGS and culture	38	50% (19/38)	20	60% (12/20)	0.5826	0.9978
Up-escalated	26		12			
De-escalated	12		8			
Adjustment by mNGS	88	22.73% (20/88)	28	35.71% (10/28)	0.2159	0.9122
Up-escalated	43		19			
De-escalated	29		9			
Adjust to antiviral treatment	2		0			
Adjust to anti-tuberculosis treatment	14		0			
No changes	43	18.60% (8/43)	18	27.78% (5/18)	0.4989	0.996
Mismatch clinical diagnosis	33	24.24% (8/33)	3	33.33% (1/3)	>0.9999	1
Adjustment by culture	4	100% (4/4)	2	50% (1/2)	0.3333	0.974
Up-escalated	3		1			
De-escalated	1		1			
Adjustment by experience	12	8.33% (1/12)	1	0% (0/1)	>0.9999	1
Up-escalated	8		1			
De-escalated	2		0			
Adjust to anti-tuberculosis treatment	2		0			
No changes	17	17.65% (3/17)	0	0% (0/0)	>0.9999	1
mNGS negative	27	22.22% (6/27)	10	10% (1/10)	0.6471	0.9981
Adjustment by experience	17	23.53% (4/17)	7	14.29% (1/7)	>0.9999	1
Up-escalated	10		6			
De-escalated	4		1			
Adjust to antiviral treatment	1		0			
Adjust to anti-tuberculosis treatment	1		0			
Adjust to antibacterial treatment	1		0			
No changes	10	20% (2/10)	3	0% (0/3)	>0.9999	1

### Analyze the influence of optimizing antimicrobial therapy on mortality rates

When considering the impact of optimizing antimicrobial therapy on mortality rates, it was noted that patients with HAS had a higher mortality rate than patients with CAS, regardless of whether treatment was optimized using mNGS co-culture results or solely mNGS results. However, this difference was not statistically significant (all adj.*P* > 0.05). Due to the small sample size, there was still no significant difference in mortality rates between CAS and HAS patients in the mNGS positive mismatch clinical diagnosis group when optimizing treatment based on traditional culture results. However, for the patients that were left, there was no significant difference in mortality rates between the two groups after accounting for antimicrobial treatment guided by clinical knowledge (Table 3).

### Comparison of medical costs between CAS and HAS

It is widely recognized that sepsis frequently manifests as a critical condition accompanied by substantial treatment expenses, thereby imposing a significant economic burden on both families and society. In order to gain deeper insights into the medical expenses of sepsis patients, this study incorporates various components of medical expenditure, including total hospitalization costs, average daily hospitalization expenses, diagnostic charges, comprehensive medical service fees, treatment expenditures, and consumables expenses. All monetary figures are denominated in US dollars. All medical expenses are expressed in US dollars, utilizing the exchange rate of Renminbi (RMB) to United States dollar (USD) as of September 3, 2023. The findings indicate that the aforementioned expenditures incurred by patients with the HAS group were considerably greater compared to those with the CAS group, and this disparity was statistically significant (all adj.*P* < 0.01). Subsequently, to assess the potential influence of mNGS results on healthcare costs, the investigation revealed no association between divergent mNGS outcomes and various medical expenditures, irrespective of the CAS or HAS group (Supplementary Tables 3–5, all adj.*P* > 0.05).

## Discussion

The guidelines set forth by the Surviving Sepsis Campaign recommend the prompt initiation of antibiotic treatment in adults deemed at risk of sepsis or septic shock, ideally within 1 h of identification (Evans et al., 2021). This practice is widely acknowledged as a crucial intervention to decrease mortality rates in septic patients, as supported by various studies (Kumar et al., 2006; Seymour et al., 2017; Bollinger et al., 2023). Factors that primarily influence the suitable antimicrobial treatment include pathogenic microorganisms, infection source (community or hospital), infection location, immune system status, existing medical conditions, local epidemiological information, and the presence of risk factors for antimicrobial resistance in patients (Gage-Brown et al., 2022). Nevertheless, the low pathogen detection rate of sepsis poses a considerable obstacle to quickly and accurately determining the cause. The impartiality of mNGS renders it highly promising for overcoming diagnostic challenges in scenarios where conventional methods may prove inadequate, such as culture-negative sepsis or polymicrobial infections. Thus, the objective of this study is to assess the efficacy and significance of mNGS in the identification of sepsis, particularly in filling the gap in pathogen detection for both community and hospital-acquired cases, with the aim of informing clinical practice and optimizing patient care.

The findings indicated that the HAS group exhibited elevated scores on the APACHE II and SOFA scales, along with an increased rate of ICU admission, prolonged hospital stays, and a higher likelihood of receiving mechanical ventilation, vasoactive drugs, blood products, and glucocorticoid treatment. These results significantly differed from those of the CAS group, aligning with previous research findings (Westphal et al., 2019; Tonai et al., 2022). The findings suggest a higher incidence of organ dysfunction in patients with HAS. Differently, our analysis identified a greater need for blood products and glucocorticoid therapy in this cohort. Additionally, our investigation uncovered manifestations of anemia, coagulation abnormalities, and renal dysfunction in the HAS group, potentially contributing to the observed distinctions. Meanwhile, our study revealed that there were no statistically significant disparities in the prevalence of past history among sepsis patients originating from CAS and HAS, suggesting that the medical history of sepsis patients remains basic consistent regardless of the infection place. We recommend that clinicians should contemplate employing similar diagnostic and therapeutic approaches for both patient cohorts.

Consistent with prior research (Duan H. et al., 2021; Sun et al., 2022), our study also found a notably elevated detection rate of mNGS in contrast to conventional culture techniques. But our research results also indicate that the high positivity rate of mNGS is not affected by the infection site, different infection sites, or sample types, which is encouraging. In addition, our study emphasizes the broader range of pathogens and higher sensitivity of mNGS in identifying sepsis-causing pathogens compared to conventional culture techniques, especially in detecting viruses and unusual pathogens. This finding underscores the robustness of mNGS in identifying pathogens, regardless of the infection site, sample type, community or nosocomial source, or etiological classification. It should be noted that antibiotic exposure may diminish the sensitivity of blood culture, whereas its impact on mNGS is minimal (Miao et al., 2018; Cheng et al., 2019). Our findings indicate that antibiotic exposure both prior to and following hospitalization, as well as varying detection opportunities, did not significantly impact the positivity rate of mNGS. However, an increase in detection frequency was observed to potentially enhance the positivity rate of mNGS, albeit without statistical significance. Thus, in the context of pathogen detection for sepsis, mNGS demonstrates a superior positive detection rate for pathogens when compared to conventional culture methods. This article posits that variables such as infection site, infection source, sample type, antibiotic exposure, and detection time have minimal influence, thereby suggesting that mNGS is a more efficient approach for pathogen identification.

As a genetic diagnostic tool, mNGS presents the added benefit of not necessitating prior screening for a specific range of etiologies during pathogen identification. This is especially advantageous when traditional culture methods fail to detect microbial agents such as *M. tuberculosis*, mycoplasma, chlamydia, and viruses in a timely manner. Previous retrospective studies have demonstrated that patients with sepsis complicated by viral infection exhibit a more severe clinical presentation and a less favorable prognosis (Duan L. W. et al., 2021). Nevertheless, the identification of viruses presents a significant obstacle for healthcare professionals. Our research revealed that Human herpesvirus was the predominant pathogen in cases of sepsis when utilizing mNGS. In the context of Human herpesviruses, populations generally display susceptibility. A thorough evaluation is necessary in a clinical setting, incorporating clinical manifestations, inflammatory markers, specific viral load quantification, as well as serum levels of immunoglobulin G and immunoglobulin M, to ascertain the existence of active infection. This finding underscores the importance of recognizing viral infection in the diagnosis and management of sepsis, warranting adequate attention. Our research indicated that bacteria, particularly Gram-negative bacilli, were predominant in the identification of sepsis pathogens through mNGS and conventional culture techniques. In cases of CAS and HAS, *K. pneumoniae* and *A. baumannii* were the bacteria most commonly identified using the methods mentioned. These results align with previous studies but highlight the superior sensitivity of mNGS over traditional culture for bacterial detection in sepsis (Geng et al., 2021; Sun et al., 2022; Qin et al., 2023; Zhou et al., 2023).

Sepsis caused by *M. tuberculosis* is commonly seen in individuals with human immunodeficiency virus (HIV), but it can also occur in those without HIV, a fact often overlooked by healthcare professionals. A delay in initiating anti-tuberculosis treatment in cases of sepsis is associated with increased mortality rates (Adegbite et al., 2023). The accurate diagnosis of *M. tuberculosis* septicemia is of significant importance, yet it is frequently misdiagnosed and overlooked in clinical practice. Our study found that mNGS method successfully identified 19 cases of *M. tuberculosis* that were missed by conventional culture methods, leading to improved detection capabilities and significantly reduced detection time. However, the lack of supporting literature necessitates further investigation through future research collaborations to confirm this finding across various patient populations and medical settings. Overall, mNGS shows distinct benefits in identifying *M. tuberculosis*. In the realm of fungal detection, traditional culture methods are characterized by their time-consuming and labor-consuming, whereas mNGS demonstrates superior sensitivity and specificity. Our research revealed that *Aspergillus* and *C. albicans* were prevalent fungi identified through mNGS and traditional culture, respectively, while mNGS also detected challenging-to-culture strains such as Pneumocystis Jirovecii and Mucoraceae. Overall, discrepancies in the strains detected by mNGS and culture techniques highlight the comprehensive and sensitive nature of mNGS, enabling the identification of elusive strains.

The advancement of detection techniques has led to a gradual rise in the identification rate of atypical pathogens, dispelling the longstanding notion that they are rare pathogen. On the contrary, these pathogens are quite prevalent and often give rise to sporadic or epidemic outbreaks. This study utilized mNGS to identify five atypical pathogens that are challenging to detect using conventional culture methods, demonstrating the efficacy of mNGS for pathogen detection. Notably, Chlamydia psittaci, a rare clinical strain, was detected through mNGS in this study. Chlamydia psittaci, a zoonotic pathogen, frequently presents with atypical symptoms resembling respiratory tract infections, such as high fever, headache, and cough, ultimately progressing to pneumonia and multi-organ failure (Zhang et al., 2020). The shortcomings of traditional etiological and serological detection techniques contribute to low positivity rates and potential misdiagnoses, underscoring the need for alternative methods like mNGS (Zhang et al., 2020; Liang et al., 2022). This research emphasizes the clinical advantages of mNGS in identifying sepsis-causing pathogens, particularly in challenging cases.

Additionally, this study observed that mNGS not only identified the types of pathogens in sepsis patients, but also revealed a higher rate of mixed pathogen infections, which contradicts the findings of traditional culture-based detection methods. Similar findings were also reported in a study investigating pathogen detection in the blood of critically ill patients, suggesting that mNGS outperforms blood culture in detecting mixed infections (Geng et al., 2021). Given these circumstances, it is imperative for clinicians to promptly identify the presence of mixed pathogen infections in sepsis patients and intervene early, as this could potentially benefit the patients. The results of the research showed significant differences in the microorganisms detected using mNGS and conventional culture techniques in cases of sepsis acquired in the community or in hospitals, especially when multiple pathogens were involved. mNGS detection exhibited a higher prevalence of bacterial combined viral among CAS and HAS, whereas traditional culture methods identified a greater number of bacterial combined fungal infections in the CAS group and multiple bacterial infections in the HAS group. These variations may be ascribed to factors such as patient origin, immune status, infection sites, local epidemic strains, and pathogen selectivity, antibiotic usage.

However, in sepsis pathogen detection, distinguishing between infection, colonization, and contamination is challenging for healthcare professionals. To reduce inaccuracies, measures like using positive and negative controls and following standard procedures are taken. Aseptic techniques were used in this study during sample collection and testing to prevent contamination. The identification of pathogenic microorganisms based on comprehensive analysis and judgment of the location of infection, microbial properties, patient symptoms, inflammatory markers, imaging findings, and inspection report (Li et al., 2023a). In the analysis of the mNGS report, pathogenic microorganisms, commensal microorganisms, and contaminants are differentiated based on criteria such as confidence level, specific sequence count, relative abundance, and coverage (Wang, 2021).

Subsequently, we conducted a more comprehensive investigation into the optimization of antimicrobial therapy based on the outcomes obtained from mNGS and/or conventional culture methods. Our findings revealed that among the 180 patients, treatment adjustments were made, with 60 patients reducing antibiotic usage, two patients discontinuing antibiotics in favor of antiviral therapy, and 14 patients transitioning to anti-tuberculosis treatment. These interventions successfully circumvented the misuse and excessive utilization of antibiotics, thereby optimizing their rational application and effectively mitigating the emergence of antibiotic resistance. Nevertheless, when considering the utilization of mNGS co-culture results vs. solely optimizing treatment with mNGS results, it was noted that the mortality rate among HAS patients was higher compared to CAS patients, but the disparity was not deemed statistically significant (all *P* > 0.05). From a clinical standpoint, the elevated mortality rate among HAS patients aligns with previous research trends (Tonai et al., 2022). Furthermore, when considering statistical power calculations, utilizing an effect size of 0.5 and a total sample size of 58 adjusted by mNGS and culture results yielded a calculated statistical power of 0.96. Similarly, with an effect size of 0.5 and a total sample size of 116 adjusted by mNGS, the calculated statistical power was 0.99. Although the observed difference did not reach statistical significance at the present sample size, the calculated statistical power indicates that the sample size may have been insufficient to detect a difference between the two groups. Therefore, the conclusion regarding the higher mortality rate in patients with HAS, while not statistically significant, may still suggest a potential trend. Notably, recent research has demonstrated contrasting findings, suggesting that tailoring antibiotic regimens using mNGS could enhance survival rates in sepsis patients (Zuo et al., 2023). The variations in research findings may be ascribed to factors such as the etiology of sepsis, location of infection, severity of the condition, sample size, and overlooking potential confounding variables such as the results of RNA detection in samples. Consequently, further inquiry is warranted to elucidate this matter.

The cost of sepsis treatment exhibits considerable variation across different countries, generally being quite high (van den Berg et al., 2022). Currently, there is a scarcity of data regarding the medical expenses associated with CAS and HAS. Our study demonstrates a significant disparity in medical costs between patients with HAS and those with CAS, with the former incurring substantially higher expenses. This statistically significant difference underscores the heavier financial burden faced by patients with HAS, thereby emphasizing the need for clinicians to prioritize the prevention of nosocomial infections. The early identification and prompt treatment of sepsis, as well as the prevention of its progression, are crucial in reducing the overall hospitalization burden associated with sepsis in a clinical setting (Paoli et al., 2018). Consequently, we conducted a comprehensive investigation into the influence of mNGS results on medical expenditures. In the context of expenses associated with sepsis treatment, mNGS does not yield substantial advantages. Given the high sensitivity of mNGS in identifying diverse bacterial species, medical professionals should consider prioritizing its early implementation over alternative approaches.

Our study is subject to certain limitations in terms of research design, interpretation of mNGS results, and evaluation of clinical value. It is crucial to mention that this research is a retrospective study carried out at one center, with a limited sample size. Consequently, while certain trends were observed in the context of CAS and HAS, statistical significance was not achieved. To address this, we plan to expand our sample size by including data from multiple centers in future research. Secondly, our findings indicate that mNGS identified a higher prevalence of mixed etiological infections in the etiological identification of sepsis. This paper solely examines the prevalent pathogen types in mixed infections, neglecting to specifically analyze the composition of mixed etiology, thereby leading to an inadequate comprehension of pathogenic microorganisms. Consequently, it is imperative to undertake further endeavors to scrutinize the precise types of mixed pathogens for enhanced sepsis treatment. Moreover, this study fails to optimize the processes of mNGS and traditional culture in the evaluation of clinical value. It does not provide evidence on the potential impact of early mNGS detection in sepsis on optimizing disease progression, reducing medical intervention and costs, and improving prognosis, further research is warranted.

## Conclusion

Overall, mNGS technology demonstrates superiority over traditional culture techniques in identifying the causative agent of sepsis, regardless of factors such as antibiotic exposure, time to detection, sampling frequency, infection site, or sample type. mNGS is particularly effective in detecting polymicrobial infections involving bacteria and viruses, enabling the identification of viral, atypical, and *M. tuberculosis* pathogens that may be overlooked by conventional cultures. Optimizing therapy with mNGS reduces antibiotic overuse without compromising prognosis. Visibly, mNGS presents distinct benefits in the realm of microbial diagnosis and antibiotic selection for sepsis, bearing significant clinical importance. The severe illness and financial burden experienced by patients with HAS underscore the necessity of infection control measures in healthcare settings. Our future research endeavors will focus on an optimization of the clinical implementation of mNGS and conventional culture techniques, with the aim of elucidating the specific effects of mNGS on sepsis outcomes, healthcare delivery, economic implications, and future prospects.

## Data availability statement

The original contributions presented in the study are included in the article/Supplementary material, further inquiries can be directed to the corresponding author.

## Ethics statement

The studies involving humans were approved by the First Affiliated Hospital of Anhui Medical University. The studies were conducted in accordance with the local legislation and institutional requirements. The participants provided their written informed consent to participate in this study.

## Author contributions

DZ: Conceptualization, Data curation, Formal analysis, Investigation, Methodology, Project administration, Software, Supervision, Validation, Writing – original draft, Writing – review & editing. XL: Data curation, Formal analysis, Software, Writing – original draft. YW: Data curation, Writing – original draft. YZ: Data curation, Writing – original draft. HZ: Conceptualization, Supervision, Writing – original draft, Writing – review & editing.
