# Supplementary material for: The clinical importance of metagenomic next-generation sequencing in detecting disease-causing microorganisms in cases of sepsis acquired in the community or hospital setting
Source: Front Microbiol. 2024 Apr 15;15:1384166. doi: 10.3389/fmicb.2024.1384166 (PMC11056561; doi:10.3389/fmicb.2024.1384166)
Supplement: Supplementary file 1 [file Data_Sheet_1.pdf]

Supplementary TABLE 1. The laboratory data of patients.

**Laboratory data**	**CAS group**	**HAS group**	***P***	**adj.*P***
WBC(x10^9/L)	8.13（5.23-13.19）	9.48（4.61-14.05）	0.8301	0.9976
Neutrophil(x10^9/L)	6.54（3.69-11.07）	7.99（2.89-12.35）	0.7003	0.9976
Lymphocyte(x10^9/L)	0.73（0.43-1.21）	0.57（0.28-1.12）	0.0145	0.2084
NLR	8.18（3.91-18.59）	10.82（3.82-20.02）	0.5847	0.9949
Hemoglobin(g/L)	102（80.50-125）	89（73-109）	0.0024	**0.0423**
RBC（x10^{12}/L）	3.38（2.66-4.16）	2.85（2.40-3.48）	0.0005	**0.0104**
Platelet(x10^9/L)	132（72.50-205.5）	99（45-175）	0.0301	0.3481
TBIL(umol/L)	14.10（9.99-24.10）	18（12.60-29.60）	0.0171	0.228
ALT(U/L)	33（19-81）	36（22-89）	0.5083	0.9931
AST(U/L)	36（24-77.50）	34（22-78）	0.7369	0.9976
Albumin(g/L)	32.50（27.75-36.40）	33.30（29.90-38.50）	0.0789	0.6066
BUN(mmol/L)	6.70（4.31-11.63）	9.60（5.83-19.73）	0.0009	**0.0178**
Scr(mmol/L)	70（50.15-120.5）	71.50（50.10-161.30）	0.7041	0.9976
PT(s)	14.60（13.55-15.80）	15.30（14-17.20）	0.0018	**0.0337**
APTT(s)	41（35.20-47.30）	42.20（37.30-48.30）	0.4315	0.9891
PTA (%)	79（68-91.90）	73（59-87）	0.0028	**0.0465**
Fibrinogen(mg/dl)	4.29（3.03-5.75）	3.71（2.67-5.28）	0.0748	0.6066
D-D(ug/L)	2.69（1.18-6.38）	2.69（1.25-6.00）	0.8765	0.9976
CRP(mg/L)	78.50（27.12-156.30）	106.2（61.90-161.10）	0.0426	0.4322
PCT(ug/L)	0.70（0.17-3.43）	0.99（0.21-6.20）	0.2802	0.9554
Lac(mmol/l)	1.82（1.36-2.39）	1.95（1.49-3.98）	0.2673	0.9554

Abbreviations: WBC, white blood cell; NLR, neutrophil to lymphocyte ratio; RBC, red blood cell; TBIL, total bilirubin; ALT, alanine aminotransferase; AST, aspartate aminotransferase; PT, prothrombin time; APTT, activated partial thrombin time; PTA, prothrombin activity; D-D, d-dimer; BUN, blood urea nitrogen; Scr, serum creatinine; CRP, C-reactive protein; PCT, procalcitonin; Lac, lactic acid.

Supplementary TABLE 2. Analyzing antibiotic exposure, timing and frequency of mNGS testing on mNGS results.

**Project**	**CAS group**			**HAS group**		
	**mNGS +**	**mNGS -**	***P*/adj.*P***	**mNGS +**	**mNGS -**	***P*/adj.*P***
**Frequency of mNGS test**			0.098<br>0.6048			0.1069<br>0.6048
1	85.88%(146/170)	14.12%（24/170）		83/33% (50/60)	16.67% (10/60)	
≥2	94.92%（56/59）	5.08%（3/59）		100%（19/19）	0% (0/19)	
**mNGS detection time**			0.699<br>0.9839			0.1619<br>0.6534
≤ 3 days	88.66%（172/194）	11.34%（22/194）		85.48%（53/62）	14.52% (9/62)	
4-7 days	90.56%（48/53）	9.43%（5/53）		100%（15/15）	0% (0/15)	
＞7 days	92.86%（39/42）	7.14%（3/42）		95.24%（20/21）	4.76% (1/21)	
**Antibiotics before admission**			0.197<br>0.6661			0.6439<br>0.9839
No	84.21% (64/76)	15.79% (12/76)		88.06% (59/67)	11.94% (8/67)	
Yes	90.20% (138/153)	9.80% (15/153)		83.33% (10/12)	16.67%（2/12）	
**Antibiotics after admission**			0.108<br>0.6048			>0.9999<br>0.9999
No	70% (7/10)	30% (3/10)		100%（4/4）	0%（0/4）	
Yes	88.64% (195/220)	11.36% (25/220)		86.67%（65/75）	13.33%（10/75）	
**Mortality rate**	27.23% (55/202)	22.22% (6/27)	0.651<br>0.9839	40.58% (28/69)	10% (1/10)	0.0828<br>0.5787

Abbreviations: mNGS+, mNGS positive; mNGS -, mNGS negative.

Supplementary TABLE 3. Comparison of medical costs between CAS and HAS groups.

**Medical costs（USD）**	**CAS group**	**HAS group**	***P***	**adj.*P***
**Total hospitalization fee**	10202（3750-25983）	27544（14235-39750）	<0.0001	0.0015
**Average daily hospitalization fee**	346.80（189.60-750）	764（470.70-1341）	<0.0001	0.0015
**Diagnosis costs**				
Laboratory diagnosis costs	1680（1127-3335）	3495（1747-5159）	<0.0001	0.0015
mNGS cost	481.6（481.6-963.2）	481.6（481.6-481.6）	<0.0001	0.0015
Laboratory diagnosis costs (excluding mNGS)	1180（680.90-2753）	3014（1421-4583）	<0.0001	0.0015
Clinical diagnosis project fee	94.53（19.88-278.50）	216.70（82.56-394.40）	0.0007	0.0015
**Integrated medical service costs**				
Medical service fee	428.60（194.60-706.60）	831.30（522.80-1186）	<0.0001	0.0015
Treatment operation fee	683.30（227.30-2598）	2184（833.80-4207）	<0.0001	0.0015
Nursing fee	292.50（109.40-989.10）	984.10（281.80-1870）	<0.0001	0.0015
Operation fee	6.88（0-55.90）	389.20（11.01-1417）	<0.0001	0.0015
**Treatment costs**				
Western medicine fee	5237（1329-12981）	12769（7755-19622）	<0.0001	0.0015
Antibacterial drug fee	1721（608.10-4410）	3819（2264-6305）	<0.0001	0.0015
Chinese patent medicine fee	16.56（0-182.60）	114（12.47-410.50）	<0.0001	0.0015
Blood products fee	0（0-250.40）	314.60（0-1099）	<0.0001	0.0015
**Consumable expenses**	361.20（107.70-1248）	2251（467.50-5687）	<0.0001	0.0015

Supplementary TABLE 4. Analyzing the impact of mNGS results on medical expenses for community-acquired sepsis.

Medical costs（USD）	CAS group		*P*	adj.*P*
	mNGS +	mNGS -		
**Total hospitalization fee**	10219 (3837-27324)	10201 (2807-22081)	0.4502	0.9962
**Average daily hospitalization fee**	344 (187.3-756.8)	356 (225-741)	0.7368	0.9987
**Diagnosis costs**				
Laboratory diagnosis costs	1676 (1141-3410)	1719 (1011-3220)	0.5246	0.9962
Laboratory diagnosis costs (excluding mNGS)	1177 (719.3-2777)	1237 (495-2739)	0.4337	0.9962
Clinical diagnosis project fee	86.5 (19-289)	114 (22-225)	0.8711	0.9987
**Integrated medical service costs**				
Medical service fee	430 (194.5-741)	408 (192-630)	0.7578	0.9987
Treatment operation fee	721 (233.3-2365)	393 (91-2761)	0.4274	0.9962
Nursing fee	293 (119.5-984.8)	285 (95-1066)	0.752	0.9987
Operation fee	5.5 (0-54.25)	8 (0-119)	0.9581	0.9987
**Treatment costs**				
Western medicine fee	5281 (1522-13605)	4133 (996-10654)	0.3292	0.9917
Antibacterial drug fee	1902 (657.5-4849)	745 (331-2919)	0.0764	0.6713
Chinese patent medicine fee	15 (0-176.8)	91 (0-248)	0.4302	0.9962
Blood products fee	0 (0-314)	0 (0-97)	0.2629	0.981
**Consumable expenses**	364 (114.5-1185)	193 (60-1753)	0.3431	0.9917

Supplementary TABLE 5. Analyzing the impact of mNGS results on medical expenses for hospital-acquired sepsis.

**Medical costs（USD）**	**HAS group**		***P***	**adj.*P***
	**mNGS +**	**mNGS -**		
**Total hospitalization fee**	27806 (15226-40888)	24727 (13269-31426)	0.3247	0.9648
**Average daily hospitalization fee**	774.8(452-1345)	591.2(434.2-1157)	0.616	0.9676
**Diagnosis costs**				
Laboratory diagnosis costs	3820(1779-5399)	2591(1614-3322)	0.0976	0.7084
Laboratory diagnosis costs (excluding mNGS)	3306(1553-4731)	2109(1133-2840)	0.0805	0.6641
Clinical diagnosis project fee	185.5(74.58-400.8)	329.8(83.08-409.4)	0.5756	0.9676
**Integrated medical service costs**				
Medical service fee	884.1(523.5-1208)	637.6(345.7-1106)	0.3105	0.9648
Treatment operation fee	2279(1090-4244)	836.8(501.4-3111)	0.0636	0.6015
Nursing fee	1030(293.9-1886)	450(242.5-1425)	0.2831	0.9641
Operation fee	616.5(11.01-1555)	116(11.01-706.6)	0.3261	0.9648
**Treatment costs**				
Western medicine fee	12769(7863-21140)	12262(6033-17429)	0.4189	0.9648
Antibacterial drug fee	3743(2250-7155)	4262(2006-6522)	0.9248	0.9676
Chinese patent medicine fee	95.03(11.19-359.9)	228.2(109.6-555.4)	0.1041	0.7084
Blood products fee	250.7(0-1130)	554.5(21.88-1143)	0.7845	0.9676
**Consumable expenses**	2311(510-6554)	1214（278.6-4921）	0.3545	0.9648

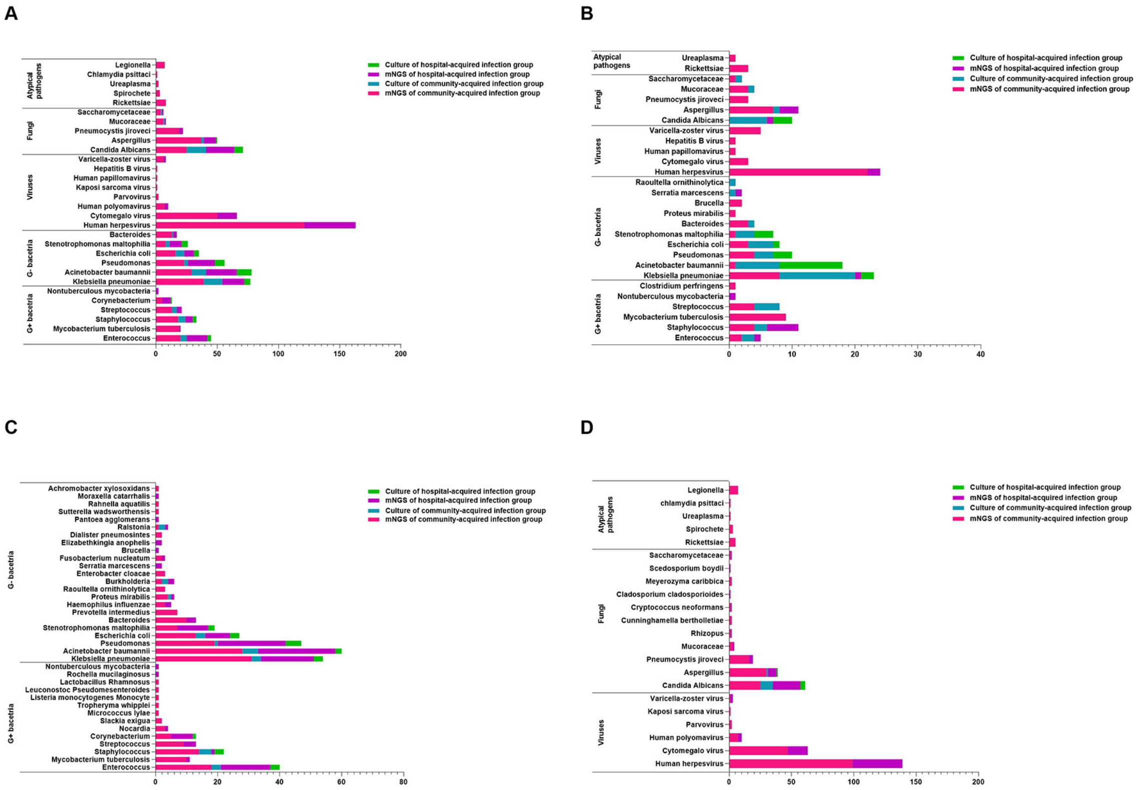

Supplementary FIGURE 1: Distribution of pathogen species by mNGS and culture. (A) Types of pathogens in CAS and HAS groups, including the top 5 Gram-positive bacteria, top 5 Gram-negative bacteria, top 5 fungi, various viruses, and atypical pathogens. (B) Single pathogens detected in CAS and HAS groups. (C) Mixed pathogens detected in CAS and HAS groups (in terms of bacteria). (D) Mixed pathogens detected by CAS and HAS groups (excluding bacteria).
